# Interleukin-15 and Soluble Interleukin-15 Receptor α in Coronary Artery Disease Patients: Association with Epicardial Fat and Indices of Adipose Tissue Distribution

**DOI:** 10.1371/journal.pone.0090960

**Published:** 2014-03-06

**Authors:** Elena Dozio, Alexis Elias Malavazos, Elena Vianello, Silvia Briganti, Giada Dogliotti, Francesco Bandera, Francesca Giacomazzi, Serenella Castelvecchio, Lorenzo Menicanti, Alexander Sigrüener, Gerd Schmitz, Massimiliano Marco Corsi Romanelli

**Affiliations:** 1 Department of Biomedical Sciences for Health, Chair of Clinical Pathology, Università degli Studi di Milano, Milan, Italy; 2 Diabetology and Metabolic Diseases Unit, I.R.C.C.S. Policlinico San Donato, San Donato Milanese, Milan, Italy; 3 Cardiology Unit, I.R.C.C.S. Policlinico San Donato, San Donato Milanese, Milan, Italy; 4 Cardiac Surgery Unit, I.R.C.C.S. Policlinico San Donato, San Donato Milanese, Milan, Italy; 5 Institute for Clinical Chemistry and Laboratory Medicine, University of Regensburg, Regensburg, Germany; 6 Service of Laboratory Medicine 1-Clinical Pathology, Department of Health Services of Diagnosis and Treatment-Laboratory Medicine, I.R.C.C.S. Policlinico San Donato, San Donato Milanese, Milan, Italy; Università degli Studi di Milano, Italy

## Abstract

Interleukin-15 (IL-15) is a pro-inflammatory cytokine which signals via a specific alpha receptor subunit (IL-15Rα). Increased IL-15 level has been observed in cardiovascular patients and IL-15 immunoreactivity has been detected at vulnerable atherosclerotic plaques. Due to the association between adipose tissue distribution, inflammation and coronary artery disease (CAD), we quantified IL-15 and IL-15Rα in CAD patients with different adiposity and adipose tissue distribution and we evaluated whether epicardial adipose tissue (EAT), a visceral fat depot surrounding and infiltrating myocardium, may be a source of both molecules. IL-15 and IL-15Rα proteins were quantified by enzyme-linked immunosorbent assays. Gene expression of IL-15 and IL-15Rα in EAT depots was evaluated by one colour microarray platform. EAT thickness was measured by echocardiography. Plasmatic IL-15 and IL-15Rα levels were higher in CAD than non-CAD patients. After classification according to adipose tissue distribution, IL-15 was higher in CAD patients with increased abdominal adiposity. Increased level of IL-15Rα was observed both in CAD and non-CAD patients with increased abdominal fat. EAT was a source of IL-15 and IL-15Rα and their expression was higher in CAD patients with increased EAT thickness. In conclusion, our data suggest that circulating levels of IL-15 and IL-15Rα seem to reflect visceral distribution of adipose tissue and that EAT may be a potential source of both IL-15 and IL-15Rα. Future studies on the relationship between IL-15, visceral fat and characteristics of atherosclerotic plaques could help to better understand the complex biology of this cytokine.

## Introduction

Interleukin-15 (IL-15) is a 14–15 kDa pro-inflammatory cytokine with the ability to induce proliferation of T cells, to enhance T and natural killer cell cytotoxicity, to protect T cells and neutrophils from apoptosis and to stimulate secretion of pro-inflammatory cytokines [1]. IL-15 signals via a trimeric membrane receptor composed by the specific IL-15R alpha chain (IL-15Rα), the IL-2R/IL15Rβ chain and the common γ chain [2]. The IL-15Rα chain may exist in several isoforms generated by alternative splicing or by proteolytic cleavage of the membrane form and it also plays an important role in transporting IL-15 outside the cells [3]–[6]. In addition to being a membrane-bound receptor, IL-15Rα is also a secreted molecule able to exert both superagonist and antagonist functions [5], [7].

Previous studies indicated that IL-15 is expressed by inflammatory cells located at vulnerable atherosclerotic plaques [8] and serum IL-15 concentration is significantly higher in patients with coronary artery disease (CAD) or peripheral artery disease than healthy people [9], [10]. Moreover, IL-15 and IL-15Rα genetic variants have been linked to an increased risk of CAD [10], [11].

The relationship between obesity, inflammation and CAD has been suggested by different studies and cumulative evidence also focused the attention on the association between visceral adipose tissue and increased risk of CAD [12]–[14]. More recently, great deal of attention has been given to epicardial adipose tissue (EAT), a metabolically active visceral adipose tissue surrounding and infiltrating myocardium and great vessels. Due to the close anatomical proximity to the heart and the absence of fascial boundaries, EAT may locally interact with myocardium and coronary arteries through paracrine secretion of pro-inflammatory and pro-atherogenic adipokines, thus suggesting that an excessive amount of EAT may represent a chronic inflammatory injury detrimental for the cardiovascular tissue and it could also play an active role in the relationship between adiposity, inflammation and cardiovascular diseases, mainly CAD [15]–[17].

Since the potential involvement of IL-15 in CAD pathogenesis and progression has been previously suggested but, to our knowledge, none of these studies have focused the attention on the contribution of adipose tissue and its distribution to CAD, in the present study we aimed: - to evaluate plasmatic levels of IL-15 and its soluble IL-15Rα in patients affected or not by CAD; - to study the relationships of IL-15 and IL-15Rα with body mass index (BMI) and indices of adipose tissue distribution [waist circumference (WC), waist to hip ratio (WHR) and EAT thickness]; - to evaluate whether EAT could also be a potential source of IL-15 and IL-15Rα.

## Materials and Methods

### Ethics Statement

The study protocol was approved by the local Ethics Committee (ASL Milano Due, n. 2516) and patients gave their written informed consent to the examination protocol, conducted in accordance with the Declaration of Helsinki, as revised in 2000.

### Study Population

A total of 82 patients undergoing open-heart surgery at the I.R.C.C.S. Policlinico San Donato (San Donato Milanese, Milan, Italy) were enrolled. According to the preoperative coronary angiographic examination, 63 were CAD patients undergoing coronary artery bypass grafting (CABG) and 19 were non-CAD patients requiring open-heart surgery for valve replacement (VR).

Patients with recent acute myocardial infarction, malignant disease, prior major abdominal surgery, renal failure, end-stage heart failure and more than 3% variation in body weight in the previous 3 months were excluded. Patients were measured for height, weight, waist and hip circumferences; body mass index (BMI) and waist/hip ratio (WHR) were calculated.

### Blood Collection

Blood samples were collected after an overnight fasting into pyrogen-free tubes with ethylendiaminetetraacetic acid as anticoagulant. Plasma samples were separated after centrifugation at 1000 g for 15 min and were stored at −20°C until analysis. Fasting glucose, insulin, total- and HDL-cholesterol, triglycerides and other biochemical parameters were assayed in the I.R.C.C.S. Policlinico San Donato central laboratory as also previously reported [18], [19]. LDL-cholesterol was calculated with the Friedewald formula. The homeostasis model assessment of insulin resistance (HOMA-IR) was calculated using the following equation: HOMA-IR = fasting insulin [µU/mL]×fasting glucose [mmol/L]/22.5.

### EAT Collection

EAT biopsy samples were harvested adjacent to the proximal right coronary artery prior to initiation of cardiopulmonary bypass pumping. Samples were stored in Allprotect Tissue Reagent (Qiagen, Hilden, Germany) at −20°C until RNA extraction.

### EAT Depot Quantification

All patients were examined by echocardiography using an M-mode color-Doppler VSF (Vingmed-System Five; General Electric, Horten, Norway) with a 2.5- to 3.5-MHz transducer probe. EAT thickness was measured on the free wall of the right ventricle from both parasternal long-and short-axis views and appears as an non-homogeneous echo-free space, as previously reported [20]. We chose to measure EAT thickness on the right ventricle for two reasons: (1) this point is recognized as the greatest absolute EAT thickness [21] and (2) parasternal long- and short-axis views allow the most accurate measurement of EAT on the right ventricle with optimal cursor beam orientation in each view [20].

### Immunoassays

Plasmatic level of IL-15 and IL-15Rα were assayed by enzyme-linked immunosorbent assays according to the manufacturer’s procedure (R&D Systems, Minneapolis, MN, USA for IL-15 and Wuhan EIAab Science, Wuhan, China for IL-15Rα).

### RNA Extraction and Gene Expression Analysis

Total RNA was extracted from tissue with the RNeasy Lipid Tissue Kit according to the manufacturer’s procedure (Qiagen, Hilden, Germany). RNA concentration was quantified by the NanoDrop 2000 (ThermoScientific, Wilmington, Germany) and RNA integrity was assessed using the Agilent RNA 6000 Nano kit and the Agilent 2100 Bioanalyzer (Agilent Technologies, Santa Clara, CA). Gene expression analysis was performed by one colour microarray platform (Agilent). 50 ng of total RNA was labelled with Cy3 using the Agilent LowInput Quick-Amp Labeling Kit-1 colour, according to the manufacturer’s instructions. cRNA was purified with the RNeasy Mini Kit (Qiagen) and the amount and labelling efficiency were measured with the NanoDrop. Hybridization was performed with the Agilent Gene Expression Hybridisation Kit, scanning was done with the Agilent G2565CA Microarray Scanner System and data were processed with the Agilent Feature Extraction software (10.7) and the ChipInspector software (Genomatix, Munich, Germany). 75 selected probes are replicated 10 times to allow intra-array reproducibility measurement.

### Statistical Analysis

Data were analyzed using GraphPad Prism 5.0 biochemical statistical package (GraphPad Software, Inc., San Diego, CA) and Statistix 7.0 (Analytical Software, Tallahassee, FL). Values of all measurements were expressed as mean ± SD and when indicated also by range, number and percentage. The normality of data distribution was assessed by the Kolmogorov-Smirnoff test. Comparison between groups was performed using two-tailed unpaired Student t test or Mann-Whitney U-test as appropriate. χ^2^ test was used for categorical variables. A multiple regression analysis was used to test the independent association of indices of fat distribution (BMI, waist circumference, EAT thickness and WHR) with IL-15 and IL-15Rα levels (dependent variables). A p value <0.05 was considered significant.

## Results

### Baseline Characteristics and Biochemical Parameters

Demographic, anthropometric and biochemical characteristics of patients are shown in table 1 and 2. Concerning anthropometric measurements, no statistically significant differences have been observed in weight, height, BMI, waist and hip circumferences between the two groups. Only WHR resulted higher in CABG than VR group (p<0.01). The mean echocardiographic EAT thickness resulted the same in the two groups. Patients with hypertension are significantly prevalent in the CABG group (p<0.001). Compared to VR, CABG patients also displayed an increased use of medications, such as aspirin (p<0.001), clopidogrel (p<0.005), β-blockers (p<0.001) and statins (p<0.01). Biochemical profile of CABG and VR patients is shown in table 2. CABG had higher levels of CRP (p<0.05), ALT (p<0.005), HbA1c (p<0.05) and lower levels of total- (p<0.005) HDL- (p<0.01) and LDL-cholesterol (p<0.05) than VR patients.

**Table 1:** Demographic and anthropometric characteristics of CABG and VR patients.

	VR (n = 19)	CABG (n = 63)	*p*
**Age (years)**	65.53±10.46 (50–86)	66.63±9.18 (48–85)	0.69
**Male sex (n, %)**	16 (84.21)	60 (95.24)	0.13
**BMI (kg/m^2^)**	27.86±3.64 (21.50–33.60)	27.51±3.97 (19.12–41.80)	0.51
**Weight (Kg)**	82.67±11.50 (56–117)	79.20±14.45 (52–135)	0.43
**Height (m)**	1.72±0.09 (1.56–1.87)	1.69±0.07 (1.52–1.85)	0.26
**Waist (cm)**	102.50±13.15 (72–122)	104.00±12.11 (72–144)	0.91
**Hip (cm)**	107.10±8.17 (98–130)	102.80±17.99 (70–210)	0.08
**WHR**	0.96±0.10 (0.68–1.10)	1.02±0.09 (0.56–1.23)	**<0.01**
**Echocardiographic EAT thickness (mm)**	7.30±2.11 (3–11)	7.85±2.45 (3–12)	0.47
**Smokers (n, %)**	7 (38.89)	35 (55.56)	0.29
**Diabetes Mellitus (n, %)**	4 (22.22)	19 (30.16)	0.57
**Hypertension (n, %)**	7 (38.89)	52 (82.54)	**<0.001**
**Family history of CAD (n, %)**	4 (22.22)	23 (36.51)	0.40
**Medications (n, %)**			
** Aspirin**	6 (33.33)	51 (80.95)	**<0.001**
** Clopidogrel**	1 (5.56)	26 (41.27)	**<0.005**
** ACEI/ARB**	9 (50.00)	41 (65.08)	0.28
** β-Blockers**	1 (5.56)	47 (74.60)	**<0.001**
**Calcium channel bloker**	1 (5.56)	16 (25.40)	0.10
** Statin**	7 (38.89)	47 (74.60)	**<0.01**

ACEI: angiotensinogen-converting enzyme inhibitor; ARB: angiotensin receptor blockade; BMI: body mass index; CABG: coronary artery bypass grafting; CAD, coronary artery disease; EAT: epicardial adipose tissue; VR: valve replacement; WHR: waist to hip ratio. Data are expressed as mean ± SD (range) or number and %.

**Table 2:** Biochemical characteristics of CABG and VR patients.

	VR (n = 18)	CABG (n = 63)	*p*
**Fasting glucose (mg/dl)**	90.60±14.28 (76–130)	99.21±41.82 (32–299)	0.71
**Fasting insulin (microU/ml)**	7.44±3.53 (1.30–13.00)	9.65±6.97 (2–45)	0.56
**HOMA-IR**	1.64±0.91 (0.29–3.08)	2.24±1.88 (0.08–10.57)	0.12
**Total cholesterol (mg/dl)**	185.20±41.73 (122–261)	148.00±34.97 (88–271)	**<0.005**
**HDL cholesterol(mg/dl)**	47.00±12.99 (23–69)	37.54±10.45 (23–78)	**<0.01**
**LDL cholesterol(mg/dl)**	110.70±38.07 (68.40–192.00)	86.00±32.44 (17.60–198.80)	**<0.05**
**Triglycerides (mg/dl)**	137.80±72.17 (81–292)	130.20±65.80 (60–367)	0.78
**Acid uric (mg/dl)**	6.64±1.39 (4.20–8.90)	6.07±1.36 (3.50–10.70)	0.18
**Creatinine (mg/dl)**	1.05±0.25 (0.63–1.51)	1.03±0.42 (0.01–2.84)	0.41
**CRP (mg/dl)**	0.20±0.16 (0.00–0.50)	1.18±2.18 (0.00–11.30)	**<0.05**
**Systolic blood pressure (mmHg)**	125.00±10.00 (110–140)	124.20±10.85 (100–160)	0.64
**Diastolic blood pressure (mmHg)**	71.67±3.89 (70–80)	71.52±5.57 (60–90)	0.89
**ALT (U/I)**	18.33±7.38 (10–32)	38.40±30.43 (8–149)	**<0.005**
**AST (U/I)**	30.53±41.50 (11–180)	31.27±31.55 (12–240)	0.94
**Bilirubin (total) (mg/dl)**	0.67±0.34 (0.34–1.59)	0.64±0.31 (0.19–1.63)	0.64
**HbA1c (%)**	4.80±1.04 (2.95–6.29)	5.62±1.27 (2.96–9.82)	**<0.05**
**Albumin (g/L)**	4.18±0.29 (3.60–4.50)	4.12±0.34 (3.30–4.90)	0.55
**Total proteins (g/L)**	6.34±0.40 (5.68–6.82)	6.38±0.63 (4.50–7.94)	0.68

ALT: alanine aminotransferase; AST: aspartate aminotransferase; CABG: coronary artery bypass grafting; CRP: C-reactive protein; HbA1C: glycated hemoglobin; HDL: high density lipoprotein;

HOMA-IR: homeostatis model of insulin resistance; VR: valve replacement. Data are expressed as mean ± SD and range.

### IL-15 Circulating Level

**Figure 1:**
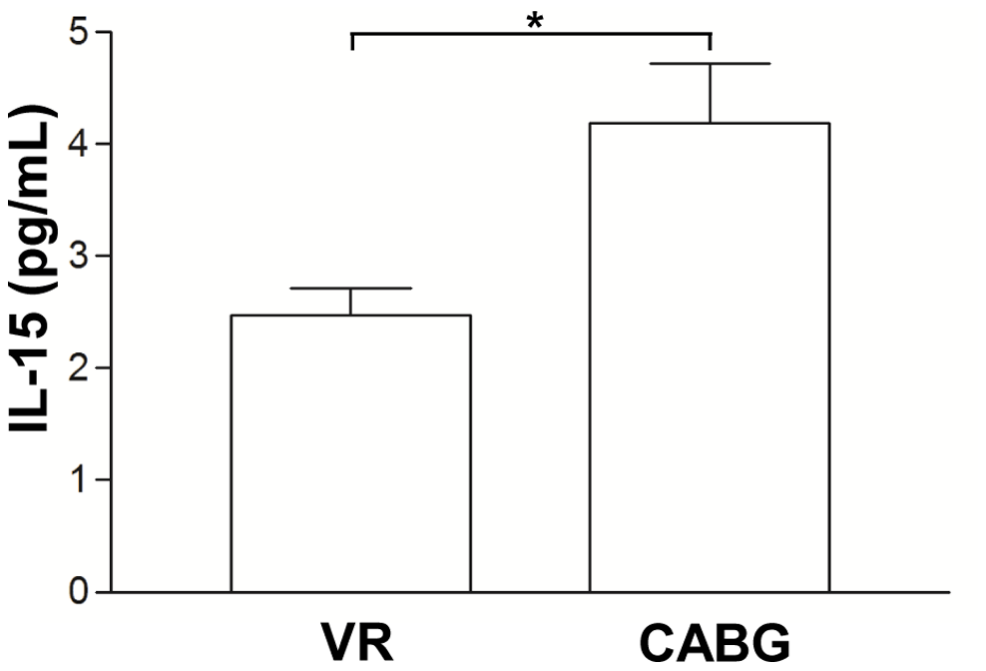
Interleukin-15 plasma level in coronary artery bypass grafting surgery (CABG) and valve replacement (VR) patients. Increased circulating level of interleukin-15 (IL-15) was observed in coronary artery bypass grafting surgery (CABG) compared to valve replacement (VR) patients. Data are mean ± SD. *p = 0.01.

Quantification of circulating IL-15 protein indicated that CABG patients had higher IL-15 level than VR patients (4.19±0.53 pg/mL *vs.* 2.48±0.24 pg/mL, p = 0.01, Figure 1).

**Figure 2:**
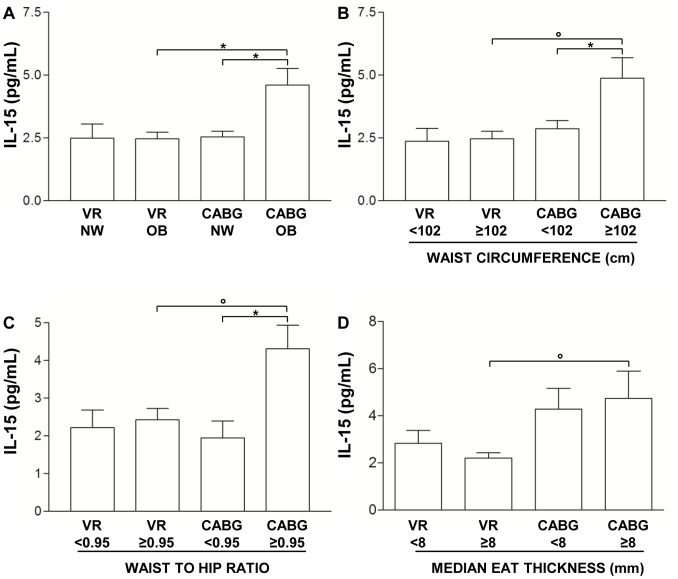
Influence of adiposity and EAT thickness on interleukin-15 plasma level. A) Interleukin-15 (IL-15) level in coronary artery bypass grafting surgery (CABG) and valve replacement (VR) patients classified, according to body mass index, in normal weight (NW) and overweight-obese (OB) groups. Data are mean ± SD; *p = 0.01. B) IL-15 level in CABG and VR patients classified, according to waist circumference cut off value of ≥102 cm. Data are mean ± SD; *p<0.05, °p<0.01. C) IL-15 level in CABG and VR patients classified according to waist-to-hip ratio (WHR) cut off value of ≥0.95. Data are mean ± SD; *p<0.05, °p = 0.01. D) IL-15 level in CABG and VR patients classified, according to the median echocardiographic epicardial adipose tissue (EAT) thickness value. Data are mean ± SD; °p = 0.01.

After patient classification according to BMI in normal weight (NW) and overweight/obese (OB) groups, we observed increased IL-15 level only in OB CABG patients (4.60±0.67 pg/mL) compared both to NW CABG (2.54±0.22 pg/mL, p = 0.01) and to OB VR patients (2.47±0.27 pg/mL, p = 0.01) (Figure 2A). No difference has been observed neither between NW VR and OB VR patients (2.50±0.56 pg/mL for NW VR *vs.* 2.47±0.27 pg/mL fro OB VR, p = 0.93), nor between NW CABG and NW VR patients (2.54±0.22 pg/mL for CABG *vs.* 2.50±0.56 pg/mL for VR, p = 0.71) (Figure 2A).

According to the different adipose tissue distribution, patients were also classified utilizing the waist circumference cut off value of ≥102 cm as a risk factor for cardio-metabolic complications in men [22]. Since 3 of the 19 VR patients and 3 of the 63 CABG patients were women, these patients were excluded from the analysis. CABG patients with a waist circumference ≥102 cm displayed higher IL-15 level (4.89±0.80 pg/mL) than CABG patients with a waist circumference <102 cm (2.87±0.32 pg/mL, p<0.05) and VR patients with a waist circumference ≥102 cm (2.47±0.30 pg/mL, p<0.01) (Figure 2B). No difference has been observed in IL-15 level between the two VR groups (2.37±0.51 pg/mL for VR <102 cm *vs.* 2.47±0.30 pg/mL for VR ≥102 cm, p = 0.86) and between CABG and VR patients with a waist circumference <102 cm (2.87±0.32 pg/mL for CABG <102 cm *vs.* 2.37±0.51 pg/mL for VR <102 cm, p = 0.57) (Figure 2B).

Another classification has been made according to the WHR cut off value of ≥0.95, for men [23]. As previously indicated for waist circumference, also for WHR classification women were excluded. CABG patients with a WHR ≥0.95 had higher IL-15 level (4.31±0.63 pg/mL) than CABG patients with a WHR <0.95 (1.95±0.45 pg/mL, p<0.05) and VR patients with a WHR ≥0.95 (2.43±0.30 pg/mL, p = 0.01) (Figure 2C). No difference has been observed in IL-15 level between the two VR groups (2.22±0.47 pg/mL for VR <0.95 *vs.* 2.43±0.30 pg/mL for VR ≥0.95, p = 0.63) and between CABG and VR patients with a WHR <0.95 (1.95±0.45 pg/mL for CABG <0.95 *vs.* 2.22±0.47 pg/mL for VR <0.95 cm, p = 0.65) (Figure 2C).

IL-15 level was also evaluated after patient classification according to the median echocardiographic EAT thickness value (8 mm for both CABG and VR groups) [19]. IL-15 level was higher in CABG patients with EAT thickness ≥ 8 mm (4.74±1.17 pg/mL) than VR patients with EAT thickness ≥ 8 mm (2.20±0.24 pg/mL, p = 0.01) (Figure 2D). No statistically significant differences have been observed in IL-15 level neither between VR groups (EAT thickness <8 mm: 2.84±0.54 pg/mL *vs.* EAT thickness ≥ 8 mm: 2.20±0.24 pg/mL, p = 0.33), nor between CABG groups (EAT thickness <8 mm: 4.29±0.88 pg/mL *vs.* EAT thickness ≥ 8 mm: 4.74±1.17 pg/mL, p = 0.86), nor between VR and CABG patients with EAT thickness <8 mm (2.84±0.54 pg/mL for VR *vs.* 4.29±0.88 pg/mL for CABG, p = 0.50) (Figure 2D).

### IL-15Rα Circulating Level

**Figure 3:**
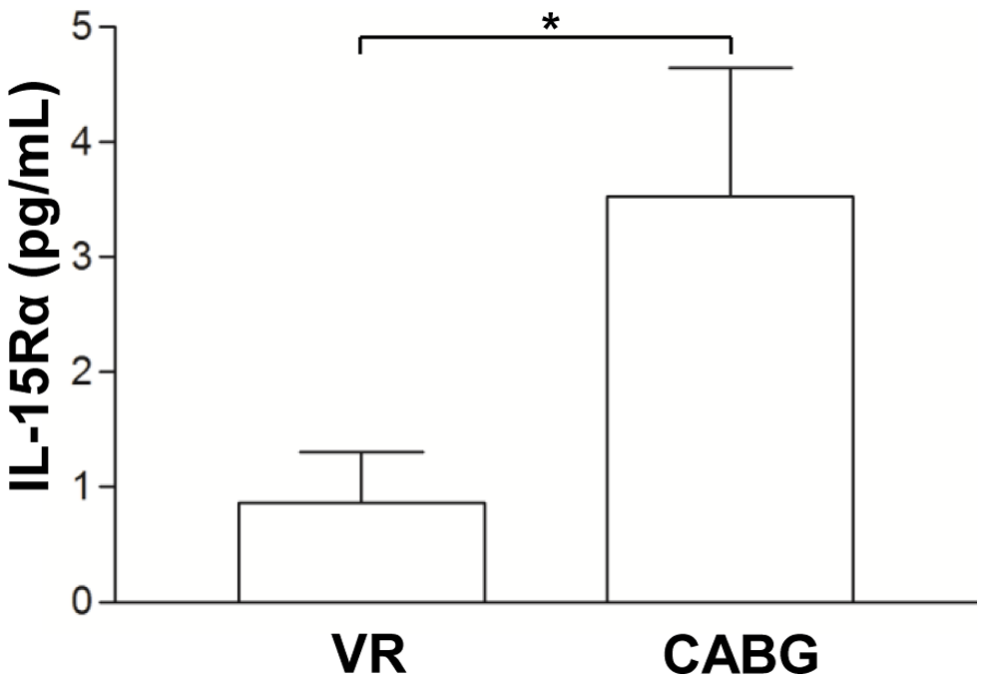
Interleukin-15 receptor α (IL-15Rα) plasma level in coronary artery bypass grafting surgery (CABG) and valve replacement (VR) patients. Increased circulating level of interleukin-15 receptor α (IL-15Rα) was observed in coronary artery bypass grafting surgery (CABG) compared to valve replacement (VR) patients. Data are mean ± SD. *p<0.05.

Quantification of circulating IL-15Rα protein indicated that CABG patients had higher IL-15Rα level than VR patients (3.53±1.12 pg/mL *vs.* 0.86±0.44 pg/mL, p<0.05, Figure 3).

**Figure 4:**
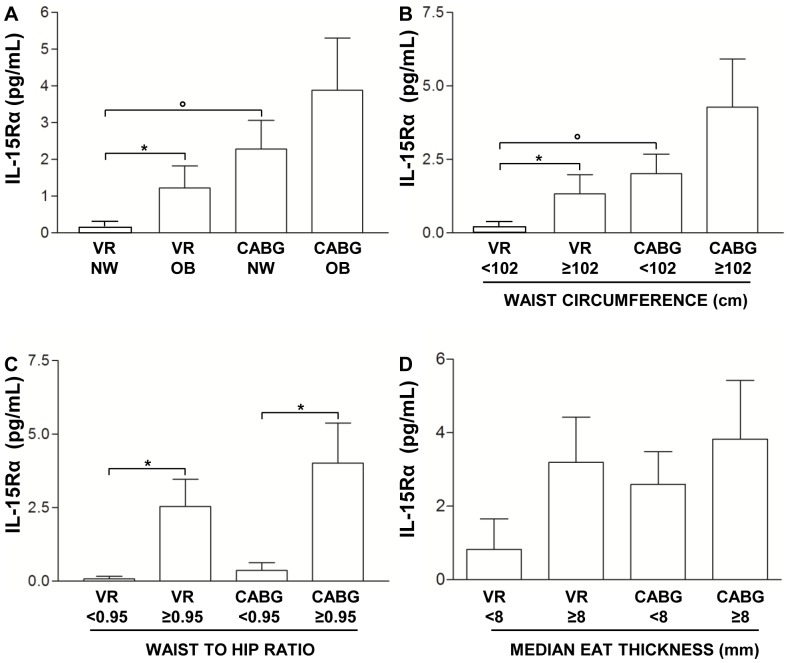
Influence of adiposity and EAT thickness on interleukin-15 receptor α plasma level. A) Interleukin-15 receptor α (IL-15Rα) level in coronary artery bypass grafting surgery (CABG) and valve replacement (VR) patients classified, according to body mass index, in normal weight (NW) and overweight-obese (OB) groups. Data are mean ± SD. *p<0.05, °p<0.001. B) IL-15Rα level in CABG and VR patients classified, according to waist circumference cut off value of ≥102 cm. Data are mean ± SD. *p<0.05, °p<0.01. C) IL-15Rα level in CABG and VR patients classified according to waist-to-hip ratio (WHR) cut off value of ≥0.95. Data are mean ± SD. *p<0.05. D) IL-15Rα level in CABG and VR patients classified according to the median echocardiographic epicardial adipose tissue (EAT) thickness value. Data are mean ± SD.

After patient classification according to BMI, OB CABG patients had higher IL-15Rα level (3.89±1.42 pg/mL) than NW CABG (2.30±0.78 pg/mL) and OB VR patients (1.23±0.60 pg/mL, p = 0.30), although both these differences did not reach the statistical significance (p>0.05 for both) (Figure 4A). A statistically significant difference has been observed between NW VR (1*10^−3^±1*10^−4^ pg/mL) and OB VR patients (1.23±0.60 pg/mL, p<0.05) and between NW VR (1*10^−3^±1*10^−4^ pg/mL) and NW CABG (2.30±0.78 pg/mL p<0.001) (Figure 4A).

According to the different adipose tissue distribution, patients were also classified utilizing the waist circumference cut off value of ≥102 cm as a risk factor for cardio-metabolic complications [22]. Since 3 of the 19 VR patients and 3 of the 63 CABG patients were women, these patients were excluded from the analysis. CABG patients with a waist circumference ≥102 cm had higher IL-15Rα level (4.28±1.64 pg/mL) than CABG patients with a waist circumference <102 cm (2.02±0.65 pg/mL) and VR patients with a waist circumference ≥102 cm (1.34±0.64 pg/mL), although both these differences did not reach the statistical significance (p>0.05 for both) (Figure 4B). A statistically significant difference has been observed between the two VR groups (1*10^−3^±1*10^−4^ pg/mL for VR <102 cm *vs*. 1.34±0.64 pg/mL for VR ≥102 cm, p<0.05) and between the groups of CABG and VR patients with a waist circumference <102 cm (2.02±0.65 pg/mL for CABG <102 cm *vs*. 1*10^−3^±1*10^−4^ pg/mL for VR <102 cm, p = 0.001) (Figure 4B).

Another classification has been made according to the WHR cut off value of ≥0.95 [23]. As previously indicated for waist circumference, also for WHR classification women were excluded. CABG patients with a WHR ≥0.95 had higher IL-15Rα level (4.02±1.36 pg/mL) than CABG patients with a WHR <0.95 (0.37±0.62 pg/mL, p<0.05) (Figure 4C). Similarly, VR patients with a WHR ≥0.95 (2.54±0.93 pg/mL) had increased level compared to VR patients with a WHR <0.95 (2.8*10^−3^±1.8*10^−3 ^pg/mL, p<0.05) (Figure 4C). No difference has been observed in IL-15Rα level neither between VR and CABG groups with a WHR <0.95 (2.8*10^−3^±1.8*10^−3 ^pg/mL *vs.*0.37±0.62 pg/mL, respectively, p = 0.30) nor between VR and CABG groups with a WHR ≥0.95 (2.54±0.93 pg/mL *vs.* 4.02±1.36 pg/mL, respectively, p = 0.37) (Figure 4C).

IL-15Rα level was also evaluated after patient classification according to the median echocardiographic EAT thickness value (8 mm for both CABG and VR groups) [19]. Although a trend of increase may be observed between VR patients with EAT thickness <8 mm (0.83±0.83 pg/mL) and ≥ 8 mm (3.19±1.23 pg/mL), between CABG patients with EAT thickness <8 mm (2.59±0.90 pg/mL) and ≥ 8 mm (3.81±1.60 pg/mL), and finally between VR and CABG groups with EAT thickness <8 mm (0.83±0.83 pg/mL vs. 2.59±0.90 pg/mL, respectively), none of these reached the statistically significance (p>0.05 for all). (Figure 4D).

### Correlates of IL-15 and IL-15Rα

The independent relations of BMI and indices of adipose tissue distribution (waist circumference, WHR and EAT thickness) with IL-15 and IL-15Rα plasma levels in CAD patients have been assessed by multiple regression analyses. WHR was the best correlate of both IL-15 (r^2^ = 0.40, p<0.001) and IL-15Rα (r^2^ = 0.39, p<0.001).

### IL-15 and IL-15Rα Gene Expression in EAT Depot Isolated from CABG and VR Patients

**Figure 5:**
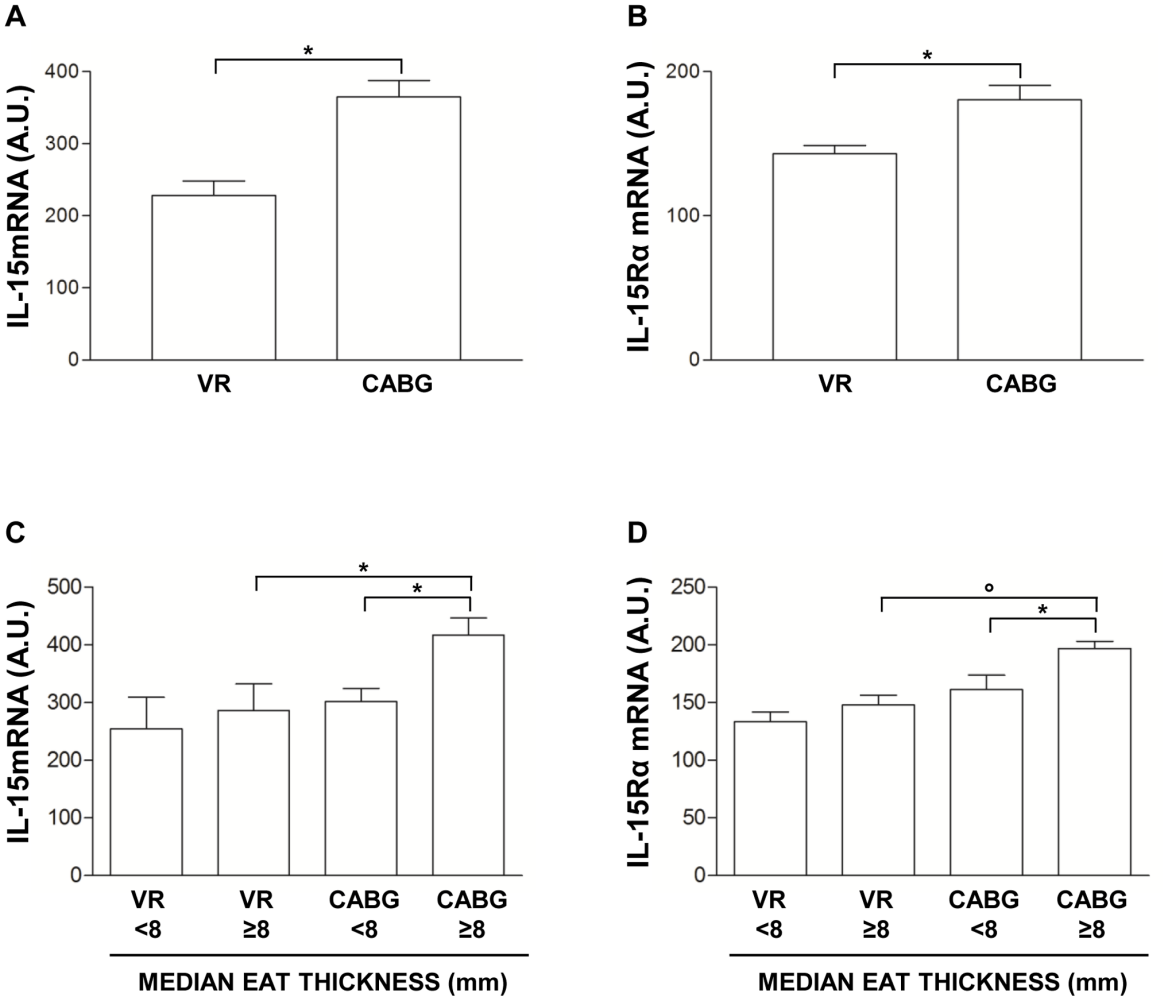
Gene expression study in epicardial adipose tissue depot. A) Quantification of interleukin-15 (IL-15) mRNA level in epicardial adipose tissue (EAT) samples obtained from patients undergoing coronary artery bypass grafting surgery (CABG) or valve replacement (VR). Data are mean ± SD. *p<0.001. B) Quantification of Interleukin-15 receptor α (IL-15Rα) mRNA level in EAT samples obtained from CABG or VR patients. Data are mean ± SD. *p<0.001. C) IL-15 mRNA level in CABG and VR patients classified according to the median EAT thickness value. Data are mean ± SD. *p<0.001. D) IL-15Rα mRNA level in CABG and VR patients classified according to the median EAT thickness value. Data are mean ± SD. *p<0.01, °p<0.001.

In CABG, IL-15 gene expression was about 1.6 time higher (p<0.001) (Figure 5A) and IL-15Rα was about 1.4 time higher than VR patients (p<0.001) (Figure 5B).

Gene expression of IL-15 and IL-15Rα was also evaluated after patient classification according to the median echocardiographic EAT thickness value (8 mm for both CABG and VR groups) [19]. CABG patients with EAT thickness ≥ 8 mm had increased IL-15 and IL-15Rα levels compared both to CABG patients with EAT thickness <8 mm (fold increase 1.4, p<0.001) and to VR patients with EAT thickness ≥ 8 mm (fold increase 1.4, p<0.001) (Figure 5C). A similar trend of increase was also observed for IL-15Rα gene. CABG patients with EAT thickness ≥ 8 mm had higher IL-15Rα level than CABG patients with EAT thickness <8 mm (fold increase 1.3, p<0.01) and VR patients with EAT thickness ≥ 8 mm (fold increase 1.3, p<0.001) (Figure 5D).

### Relationship between Epicardial Fat Thickness and Indexes of Adipose Tissue Distribution (Waist Circumference and WHR)

**Figure 6:**
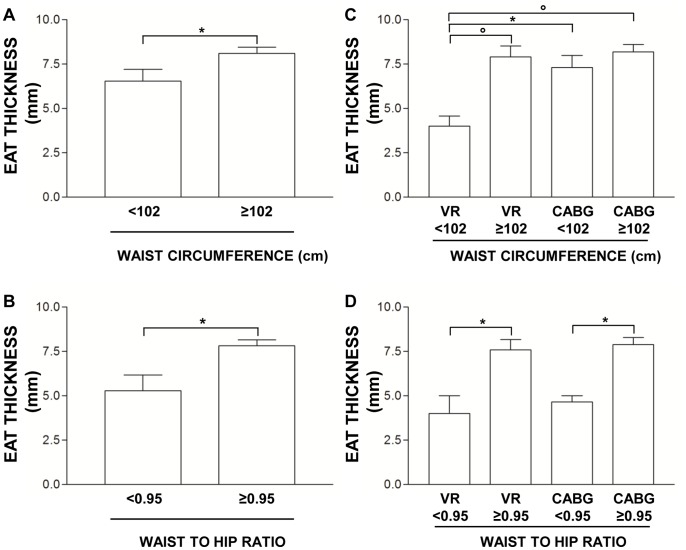
Influence of indices of adipose tissue distribution on EAT thickness. A) Echocardiographic quantification of epicardial adipose tissue (EAT) thickness in all cardiovascular patients (CABG+VR) classified according to waist circumference cut off value ≥102 cm. Data are mean ± SD. *p<0.05. B) Echocardiographic quantification of EAT thickness in all cardiovascular patients (CABG+VR) classified according to waist-to hip ratio cut off value ≥0.95. Data are mean ± SD. *p<0.05. C) Echocardiographic quantification of EAT thickness in CABG and VR classified according to waist circumference cut off value ≥102 cm. Data are mean ± SD. *p<0.05, ° p<0.01. D) Echocardiographic quantification of EAT thickness in CABG and VR patients classified according to waist-to hip ratio cut off value ≥0.95. Data are mean ± SD. *p<0.05.

All cardiovascular patients (CABG+VR) were first classified according both to waist circumference cut-off value of ≥ 102 cm and WHR ≥ 0.95 and then echocardiographic EAT thickness was evaluated in each subgroup. Patients with a waist circumference ≥ 102 cm had higher EAT thickness (8.12±0.35 mm) than patients with a waist circumference <102 cm (6.50±0.67 mm, p<0.05) (Figure 6A). Similarly, patients with a WHR ≥ 0.95 displayed increased EAT thickness (7.82±0.33 mm) compared to patients with a WHR <0.95 cm (5.29±0.89 mm, p<0.01) (Figure 6B).

We next performed the same analysis after patients classification in CABG and VR groups. VR patients with a waist circumference ≥ 102 cm had higher EAT thickness (7.91±0.61 mm) compared to VR patients with a waist circumference <102 cm (4.00±0.58 mm, p<0.01). We also observed higher EAT thickness in CABG patients with a waist circumference <102 cm (7.30±0.68 mm) than VR patients with the same waist circumference (4.00±0.58 mm, p<0.05). In CABG patients with a waist circumference ≥ 102 cm, EAT thickness (8.12±0.42 mm) was higher only compared to VR patients with a waist circumference <102 cm (4.00±0.58 mm, p<0.01), whereas it was not different neither from CABG patients with a waist circumference <102 cm (7.30±0.68 mm, p = 0.30) nor from VR patients with a waist circumference ≥ 102 cm (7.91±0.61 mm, p = 0.73) (Figure 6C).

Relative to WHR, EAT thickness resulted higher both in VR (7.60±0.58 mm) and CABG patients (7.89±0.40 mm) with a WHR ≥ 0.95 compared to VR (4.00±1.00 mm, p<0.05) and CABG patients (4.67±0.33 mm, p<0.05) with a WHR <0.95, respectively (Figure 6C). No differences have been observed neither between VR (4.00±1.00 mm) and CABG (4.67±0.33 mm, p = 0.50) patients with a WHR <0.95, nor between VR (7.60±0.58 mm) and CABG patients (7.89±0.40 mm, p = 0.73) with a WHR ≥ 0.95 (Figure 6C).

## Discussion and Conclusion

In the present study, plasmatic IL-15 and IL-15Rα levels were significantly higher in CABG compared to VR patients. Anyway, after patient classification according to BMI and indices of adipose tissue distribution, we observed that IL-15 increase is limited to the subgroup of overweight-obese CABG patients and, in particular, to those patients displaying increased abdominal adiposity. Increased IL-15Rα level was present, instead, both in CABG and VR patients with increased abdominal fat accumulation. Moreover, we demonstrated, for the first time, that EAT depot is a potential source of IL-15 and IL-15Rα and that gene expression of both genes is higher in EAT depot isolated from CABG than VR patients, and, among CABG, in patients with increased EAT thickness.

It is well known that a systemic inflammatory response might be involved in the occurrence of CAD and that obesity, mainly visceral obesity, is associated with an inflammatory and atherogenic profile [14]. In obesity, adipose tissue develops an inflammatory environment due to infiltrating macrophages which are a source of numerous pro-inflammatory cytokines [24]. From this point of view there have been several reports discussing the potential role of different cytokines (such as IL-1β, IL-6, IL-8, TNFα) in the development of cardiovascular diseases (reviewed in [25]), but, to our opinion, little attention has been given to IL-15 in this relationship.

Previous *in vitro* studies indicated that both pre-adipocytes and differentiated adipocytes do not significantly express IL-15 [26]. Anyway, till now, no studies on IL-15 expression in various adipose depots and in different physiological/pathological conditions have been performed. It is also unknown whether the obesity-associated inflammation could stimulate IL-15 expression by adipocytes or other cell types within the adipose tissue or in other tissues. Our data suggest that the obesity status of the patients may be involved in increasing IL-15 expression only in patients displaying a yet activated detrimental inflammatory process, such as atherosclerosis in CAD patients. In fact, the obesity-related increase of IL-15 at both plasmatic and EAT level has not been observed in overweight-obese VR patients. Moreover, since Houtkamp et al. [8] have shown that IL-15 immunoreactivity is expressed by the majority of macrophages in both lipid-rich and fibrolipid plaques, but not in normal vessels, it is possible that the chronic low-grade inflammatory status associated to obesity may promote the locally expression of the cytokine just in CAD patients. Our results empathize also the role of fat accumulation at abdominal level in promoting IL-15 increase. In fact, after patient classification according to WHR, which is recognize as a worldwide index of visceral adipose tissue distribution [22], the significant up-regulation of IL-15 level previously observed after BMI classification remained still present and WHR resulted also the best independent correlate of IL-15. Although we demonstrated that the co-presence of visceral obesity and CAD is crucial in promoting IL-15 up-regulation, anyway our data did not prove what could be the main source of the cytokine. Here, we have observed for the first time that the visceral EAT expresses the cytokine and that IL-15 is produced at higher level in the subgroup of CAD patients with increased EAT thickness. This observation seems to suggest that, as previously observed at plasmatic level, also at EAT level IL-15 up-regulation strongly reflects the co-presence of local fat deposition and CAD. In a previous paper Iacobellis et al. [27] indicated that there is a direct relationship between the amount of EAT and the severity of CAD and that a mass-dependent mechanism might be evoked to explain the increased activity of EAT in CAD. In addition, other studies also suggested that EAT adjacent to CAD exhibited infiltration by chronic inflammatory cells [15] and that the expression of many adipokines and cytokines genes in EAT is related to CAD severity [28]. Although of novelty, our result on IL-15 expression may be considered only preliminary. In fact, due to the lack of data on coronary stenosis degree, on the light of previous papers [15], [28] we may here also suppose that IL-15 expression at EAT level is increased according to CAD severity. Moreover, whether EAT also secretes the cytokine and directly contributes to increase IL-15 plasma level remains to be evaluated.

Bergamaschi et al., by using a self-made ELISA assay able to specifically measure the IL-15/IL-15Rα complex, suggested that both in human and mouse serum IL-15 is poorly secreted and unstable and that the amount of IL-15/IL-15Rα complex was similar to the total IL-15 quantified by a commercial IL-15 ELISA assay able to measure both the single chain and the heterodimer [29]. Since in our study the quantification of IL-15 has been performed by a commercial kit able to recognize the cytokine both as single chain and complex, this previous observation by Bergamaschi prompted us to consider that what we have really measured was not the single and unstable IL-15 chain but the more stable IL-15/IL-15Rα complex, which may play like an hormone. The scenario of IL-15 signalling is further complicated by the possibility that free circulating IL-15Rα may interact with IL-15 retained at the cellular membranes through an IL-15R-independent mechanism [30], [31]. For these reasons, in our study we decided to quantify the free circulating form of IL-15Rα, by utilizing a specific kit able to recognize the soluble free receptor, with the aim to explore also the potential contribution of this form in modulating IL-15 biological functions. Differently from IL-15, after patients classification according to BMI, waist circumference and WHR, we observed an up-regulation of IL-15Rα level not only between the two subgroups of CABG but also VR patients. To be noted, this difference appears more clearly defined when patients were classified according to WHR and WHR resulted also the best independent correlate of IL-15Rα. To our opinion these observations suggest that IL-15Rα up-regulation is mainly related to the amount of visceral adipose tissue, and that the inflammatory status associated to visceral obesity may be directly involved in stimulating its increase via the activation of NF-kappaB, as previously observed [32]. Although the presence of CAD seems not to play a major role in promoting plasma IL-15Rα increase, independently of visceral obesity, the up-regulation of the cytokine expression at EAT appears to be related to the pathology, instead. In fact, as previously discussed for IL-15, also IL-15Rα up-regulation strongly reflects the co-presence of both local increased fat deposition and CAD. This could suggests that CAD may affect local cytokine expression at EAT. Whether EAT also secretes the cytokine and directly contributes to increase IL-15 plasma level remains to be evaluated.

One important limit of the study is the absence of SAT to compare the expression of IL-15 and IL-15Rα between different types of adipose tissues (visceral -EAT- *vs.* subcutaneous). This could be an important issue to address in future studies to clarify whether, in the presence of CAD, the expression of IL-15 and IL-15Rα is increased only at EAT level or other fat depots may be important sources of both molecules. A second limit of our study may be the fact that, presently, we performed only a gene expression study and we did not directly quantify the amount of both IL-15 and IL-15Rα proteins produced by EAT depot. Since isolation of EAT during surgery is a delicate and difficult procedure and the amount of tissue isolated is often poor, in particular in the case of normal weight patients, and not enough to perform both RNA and protein expression analysis, we first decided to carry out a gene expression study. A third limit of our study is that, due to the immunoreactivity features of the assay utilized, we are not able to discriminate the single contribution of each of the two previously described IL-15Rα variants: one arising from alternative splicing and not containing the transmembrane domain and the other arising from proteolytic cleavage of membrane receptor and containing the transmembrane domain [26]. Since different effects have been described for the two variants, future studies exploring potential differences among the two forms could help to better clarify the pathophysiological role of IL-15Rα in the field of visceral adiposity and CAD.

In conclusion, our data indicate that adipose tissue distribution may play a role in increasing circulating IL-15 and IL-15Rα levels and that EAT, a visceral fat depot surrounding the myocardium, may be a potential source of IL-15 and IL-15Rα. Future studies aimed to evaluate a potential correlation between circulating IL-15 level and plaques characteristics and the evaluation of circulating IL-15Rα forms could help to better understand the complex biology of this cytokine in the field of adiposity and CAD.
